# *LIG1* is a novel marker for bladder cancer prognosis: evidence based on experimental studies, machine learning and single-cell sequencing

**DOI:** 10.3389/fimmu.2024.1419126

**Published:** 2024-08-21

**Authors:** Ding-ming Song, Tong Shen, Kun Feng, Yi-bo He, Shi-liang Chen, Yang Zhang, Wen-fei Luo, Lu Han, Ming Tong, Yanyang Jin

**Affiliations:** ^1^ Department of Urology, Jinzhou Medical University, The First Hospital of Jinzhou Medical University, Jinzhou, Liaoning, China; ^2^ Department of Clinical Lab, The First Affiliated Hospital of Zhejiang Chinese Medical University (Zhejiang Provincial Hospital of Chinese Medicine), Hangzhou, Zhejiang, China; ^3^ Department of Vascular Surgery, Fuwai Yunnan Cardiovascular Hospital, Affiliated Cardiovascular Hospital of Kunming Medical University, Kunming, Yunnan, China; ^4^ Jinzhou Medical University, The First Hospital of Jinzhou Medical University, Jinzhou, Liaoning, China; ^5^ Department of Infectious Diseases, The Affiliated Hospital of Guizhou Medical University, Guiyang, Guizhou, China

**Keywords:** *LIG1*, urothelial bladder cancer, single-cell, machine-learning, bioinformatics, tumorinfiltrating immune cell

## Abstract

**Background:**

Bladder cancer, a highly fatal disease, poses a significant threat to patients. Positioned at 19q13.2-13.3, LIG1, one of the four DNA ligases in mammalian cells, is frequently deleted in tumour cells of diverse origins. Despite this, the precise involvement of LIG1 in BLCA remains elusive. This pioneering investigation delves into the uncharted territory of LIG1’s impact on BLCA. Our primary objective is to elucidate the intricate interplay between LIG1 and BLCA, alongside exploring its correlation with various clinicopathological factors.

**Methods:**

We retrieved gene expression data of para-carcinoma tissues and bladder cancer (BLCA) from the GEO repository. Single-cell sequencing data were processed using the “Seurat” package. Differential expression analysis was then performed with the “Limma” package. The construction of scale-free gene co-expression networks was achieved using the “WGCNA” package. Subsequently, a Venn diagram was utilized to extract genes from the positively correlated modules identified by WGCNA and intersect them with differentially expressed genes (DEGs), isolating the overlapping genes. The “STRINGdb” package was employed to establish the protein-protein interaction (PPI) network.Hub genes were identified through the PPI network using the Betweenness Centrality (BC) algorithm. We conducted KEGG and GO enrichment analyses to uncover the regulatory mechanisms and biological functions associated with the hub genes. A machine-learning diagnostic model was established using the R package “mlr3verse.” Mutation profiles between the LIG1^high and LIG1^low groups were visualized using the BEST website. Survival analyses within the LIG1^high and LIG1^low groups were performed using the BEST website and the GENT2 website. Finally, a series of functional experiments were executed to validate the functional role of LIG1 in BLCA.

**Results:**

Our investigation revealed an upregulation of LIG1 in BLCA specimens, with heightened LIG1 levels correlating with unfavorable overall survival outcomes. Functional enrichment analysis of hub genes, as evidenced by GO and KEGG enrichment analyses, highlighted LIG1’s involvement in critical function such as the DNA replication, cellular senescence, cell cycle and the p53 signalling pathway. Notably, the mutational landscape of BLCA varied significantly between LIG1^high^ and LIG1^low^ groups.Immune infiltrating analyses suggested a pivotal role for LIG1 in immune cell recruitment and immune regulation within the BLCA microenvironment, thereby impacting prognosis. Subsequent experimental validations further underscored the significance of LIG1 in BLCA pathogenesis, consolidating its functional relevance in BLCA samples.

**Conclusions:**

Our research demonstrates that LIG1 plays a crucial role in promoting bladder cancer malignant progression by heightening proliferation, invasion, EMT, and other key functions, thereby serving as a potential risk biomarker.

## Graphical Abstract

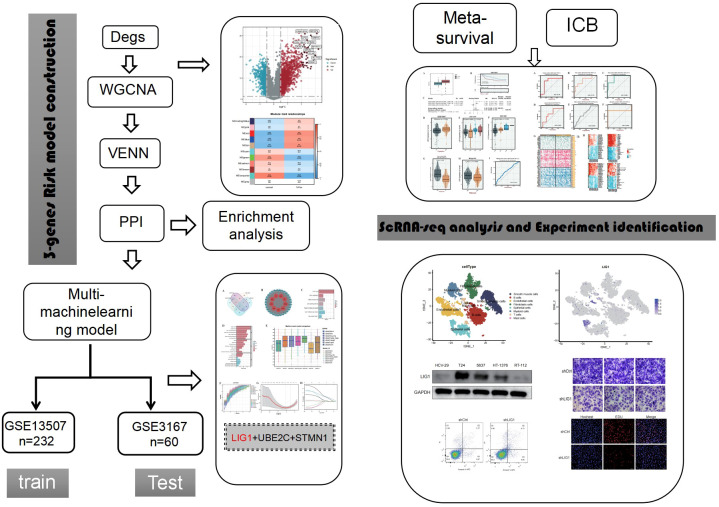

## 1 Introduction

Bladder cancer is the most common malignant tumour in the urinary system, with approximately 900,000 new cases diagnosed every year (1, 2). The characteristics of bladder cancer are a multifocal growth pattern and high rate of recurrence (3); therefore, non-muscular invasive bladder cancer often poses a heavy public health burden (4). Patients who underwent radical resection were reported to have the longest median survival time of approximately 48 months (5). Radical cystectomy and cisplatin-based chemotherapy are standard treatments; however, there is currently no significant progress in treatment efficacy. The use of immune checkpoint inhibitor (ICI) therapies, such as anti-PD1 therapy, has resulted in a breakthrough in the treatment of urothelial cell carcinoma in recent years (6, 7). However, due to the strong heterogeneity of bladder cancer, its efficacy is still very limited at approximately 30% (7). Therefore, at present, the overall treatment effect of bladder cancer is not satisfactory, and new treatment breakthroughs are urgently needed (8).

Currently, with the advancement of bioinformatics technology and machine learning, research strategies for urothelial cell carcinoma are becoming increasingly diverse (9, 10). On the one hand, clustering analysis and other techniques can be used to identify subgroups that may be more sensitive to immunotherapy or chemotherapy, to avoid ineffective clinical treatment and achieve precise or personalised treatment (11, 12). On the other hand, machine learning and single-cell analysis are used to search for key genes and identify new therapeutic targets (13, 14). In our study, we identified key genes associated with bladder cancer by combining the advantages of highly variable genes, weighted gene co-expression network analysis (WGCNA), machine learning, and single-cell sequencing. We also validated the regulatory role of LIG1 in bladder cancer cells, as well as its potential as a biomarker and its therapeutic value through cell function experiments.

## 2 Materials and methods

### 2.1 Acquisition of data

Gene expression profiles were acquired from the Gene Expression Omnibus (GEO) database (www.ncbi.nlm.nih.gov/geo). Specifically, the BLCA dataset GSE13507 included 67 adjacent normal tissue samples and 165 *in situ* bladder cancer tissue samples, excluding recurrent bladder cancer samples. Additionally, dataset GSE3167 consisted of 14 normal bladder tissue samples and 46 *in situ* bladder cancer tissue samples. GSE13507 served as the training set for machine learning, while GSE3167 was utilised as the external validation set. The control group comprised all adjacent normal tissue samples and normal bladder tissue samples, while the cancer group consisted of *in situ* bladder cancer tissue samples. The gene expression profile arrays of GSE13507 and GSE3167 were based on the GPL6102 platform (Illumina human-6 v2.0 expression bead chip) and GPL96 platform ([HG-U133A] Affymetrix Human Genome U133A Array), respectively. Single-cell transcriptome data were sourced from GSA-Human ID HRA000212 and 11 bladder cancer fastq files labelled SRR12603780 to SRR12603790 in the BioProject database, including three single-cell samples of normal bladder tissue and eight single-cell samples of *in situ* bladder cancer.

### 2.2 Preparation of data

The gene expression data from bladder urothelial carcinoma datasets underwent processing using R software (version 4.2.2) and Bioconductor Packages. Initially, the transcriptome files were standardised by converting probe IDs to IDs through platform files, before transforming them into FPKM format files. Subsequently, Cell Ranger (version 7.0.0) was utilised to handle the raw data, address multiple barcodes, and to interpret the transcriptome maps and sub-sample reads to generate normalised summary data across the samples. This process yielded a raw unique molecular identifier (UMI) count matrix, which was further converted to a Seurat object using the R package Seurat (version 4.3.0). Cells with UMI counts <1000 or with mitochondrial-derived UMI counts exceeding 20%, along with cells exhibiting erythrocyte-derived UMI counts surpassing 20%, were identified as low-quality cells and were subsequently eliminated. Following quality control, the UMI count matrix underwent log-normalisation, and batch effects were mitigated using sample IDs. The top 2000 variable genes were chosen for the downstream analysis, and the harmony function was applied for normalisation to eliminate potential batch effects. Principal component analysis (PCA) was conducted on the integrated data matrix to reduce the dimensionality, with the top 20 principal components selected for subsequent analysis utilising Seurat’s Elbowplot function. The FindClusters function in Seurat was employed to recognise major cell clusters with a resolution of 2.5. The data was visualised through two-dimensional tSNE or UMAP plots. Established conventional markers were utilised to initially categorise the cells into seven major cell types. Each major cell type was further subdivided into subsets and subclustered to identify heterogeneity within each cell type. The Seurat FindAllMarkers function was utilised to pinpoint genes that were preferentially expressed or differentially expressed in tumour and normal-derived cells.

### 2.3 Differentially expressed genes and WGCNA

In our investigation involving bladder cancer patients, we conducted a comparative analysis between the cancer tissue and adjacent normal tissue to identify DEGs. The “limma” package was employed with filtering criteria logFC > 0.5 and p < 0.05 to pinpoint these genes, which were subsequently presented visually. Additionally, WGCNA was carried out on the entire gene set; the analysis was performed separately on the two datasets to construct unsigned co-expression networks aimed at detecting co-expression modules. Initially, sample stratification clustering analysis was executed using the flashClust tool in R to detect and remove outlier samples. Subsequently, a biologically relevant scale-free network was established based on the scale-free topology criterion utilising the “pickSoft-Threshold” algorithm in WGCNA to determine the appropriate “soft” threshold power (β). Next, a Topological Overlap Matrix (TOM) was generated from the adjacency matrix, and gene modules were identified through the dynamic tree-cut algorithm. Gene significance (GS) and module membership (MM) were then computed to correlate modules with clinical characteristics, and the network of feature genes was visually represented.

### 2.4 Somatic mutations and copy number variations

Bladder cancer somatic mutation data and CNV data from The Cancer Genome Atlas (TCGA) database were analysed on the BEST website (https://rookieutopia.com/app_direct/BEST); images were created on the website using maftools and GISTIC2.0. The visualisation of mutations was conducted according to varying levels of *LIG1* gene expression.

### 2.5 Protein–protein interactions

The R package “STRINGdb” (version 2.8.4) was employed with a confidence threshold of 400(equivalent to a 0.4 confidence score) to establish a PPI network.By applying Betweenness Centrality (BC), we identified common genes within the network, with a focus on the genes exhibiting the highest levels of interaction. Genes with higher BC values were considered as hub genes, as they play a crucial role in the connectivity and information flow within the network. These hub genes are likely to have a significant impact on disease comorbidity. Subsequently, the PPI network was visualised using Cytoscape (version 3.9.1) software.

### 2.6 Enrichment

The nine hub genes discovered in the PPI network analysis underwent further analysis to explore their distinctive biological and functional features through Gene Ontology (GO) and Kyoto Encyclopedia of Genes and Genomes (KEGG) pathway analysis using the clusterProfiler package (version 4.6.2) and the DOSE package (version 3.24.2). Significance was determined by p < 0.05, with a greater GeneRatio indicating increased significance.

### 2.7 Machine learning

Using the R package “mlr3verse,” a machine learning diagnostic model was established with the dataset GSE13507 and matched clinical data. A validation model was then built using GSE3167 and corresponding clinical data. Various machine learning modelling techniques were employed, and the ranger model with the best performance was selected to estimate the significant role of the hub genes in disease diagnosis. Subsequently, a Least Absolute Shrinkage and Selection Operator (LASSO) regression model was used to identify the top genes among the hub genes based on their importance proportions.

### 2.8 Survival analysis

Utilising the GENT2 website (http://gent2.appex.kr/gent2), survival analysis was performed on the key genes by the meta-survival evaluation. Subsequent validation of the influence of key genes on various survival rates according to tumour TNM staging and progression was conducted on the BEST website (https://rookieutopia.com/app_direct/BEST). Clinical data from the TCGA and GEO databases were utilised in the analysis performed on both online platforms.

### 2.9 Immune infiltration analysis

On the BEST website (https://rookieutopia.com/app_direct/BEST), the immune infiltration status of bladder cancer with high *LIG1* expression was comprehensively evaluated using the CIBERSORT, CIBERSORT_ABS, EPIC, ESTIMATE, MCPcounter, Quantiseq, TIMER, and xCell algorithms. The analysis utilised the following datasets: GSE154261, GSE39281, GSE52219, GSE70691, GSE37815, GSE48276, GSE69795, GSE19423, GSE48075, IMvigor210, GSE13507, TCGABLCA and GSE31684.

### 2.10 Drug targeted therapy

Targeted therapy analysis of genes was conducted using the BEST website. High expression of key genes was predicted for their immune response to platinum-based chemotherapy, PD-1 ICI therapy, PD-L1 ICI therapy, CTLA-4 ICI therapy, and CAR-T therapy using the IMvigor210 cohort, Amato cohort, Kim cohort, Gao cohort, Lauss cohort, Riaz cohort, and Ascierto cohort.

### 2.11 Candidate drug prediction

Based on the drug prediction analysis queues (1. GDSC_V1 2. GDSC_V2 3. CTRP 4. PRISM) on the BEST website (https://rookieutopia.com/app_direct/BEST), possible bladder cancer targeted therapy drug information and ICI analysis were predicted using the following datasets: GSE37815, GSE154261, GSE13507, GSE19423, TCGABLCA, GSE31684, GSE48276, GSE69795, IMvigor210, GSE70691, GSE48075, GSE39281, and GSE52219.

### 2.12 Single-cell sequencing

In R language, using the “Seurat” package, we performed dimensionality reduction clustering on single-cell sequencing data. We identified 34 clusters with a resolution of 2.5 and determined the expression levels of feature genes in these 34 clusters. Specifically, we identified mast cells (TPSAB1+), T cells (CD3D+), myeloid cells (LYZ+), epithelial cells (EPCAM+), fibroblasts (LUM+), endothelial cells (VWF+), B cells (CD79A+) and smooth muscle cells (ACTA2+) within these clusters. Using the “FindAllMarkers” function in the single-cell sequencing dataset, we determined the expression and localisation of the *LIG1* gene. Subsequently, we conducted enrichment analysis on each cluster using the “GSVA” package and explored cell–cell interactions among the different cell clusters using the “CellChat” package.

### 2.13 Cell culture

Bladder cancer cell lines (T24, HT-1376, RT-112, and 5637) and the human normal bladder epithelium cell line HCV-29 were obtained from the Cell Bank of the Chinese Academy of Sciences located in Shanghai, China. These cells were all maintained in RPMI-1640 medium (Gibco, USA) enriched with 10% foetal bovine serum (FBS; Gibco, USA). The culture conditions were set at 37°C in a humidified incubator with 5% CO_2_.

### 2.14 Cell transfection

The lentivirus used for *LIG1* suppression was procured from OBIO (GENECHEM, Shanghai). T24 bladder cancer cells were cultured in 6-well dishes until they reached 60% confluence. The cells were then infected with either the *LIG1* knockdown lentivirus (also known as *shLIG1*) or a scramble control (known as *shCtrl*). Stable transduction pools were created by selecting with puromycin (2 μg/ml) over a 2-week period. The efficiency of the transfection was verified using quantitative reverse transcription polymerase chain reaction (qRT-PCR) and Western blotting analyses.

### 2.15 qRT-PCR

Total RNA was extracted from cultured cells using Trizol reagent (Beyotime, Shanghai, China), followed by cDNA synthesis utilising NovoScript^®^ Plus 1st Strand cDNA Synthesis SuperMix (Novoprotein Scientific Inc., Shanghai, China). Subsequently, qRT-PCR was conducted with SYBR High-Sensitivity qPCR SuperMix (Novoprotein Scientific Inc., Shanghai, China), and the transcriptional levels were normalised to the internal control gene, *GAPDH*. The primer sequences utilised were as follows: *GAPDH* forward 5′-ATCATCAGCAATGCCTCC-3′ and reverse 5′-CATCACGCCACAGTTTCC-G-3′; *LIG1* forward 5’-CCCATCGGTCACATCCTT-3’ and reverse 5’-ATCCACCTCCTTGCGTTT-3’. The relative expression levels of the target gene were determined using the 2^-ΔΔCT^ method.

### 2.16 Western blotting

Cells were collected and lysed with RIPA buffer (Beyotime, Shanghai, China), which was enhanced with the protease inhibitor PMSF (Beyotime, Shanghai, China). The protein concentration was ascertained using a BCA protein quantitative kit (Beyotime, Shanghai, China). A total of 30 μg of each protein sample was separated using 10% sodium dodecyl sulphate-polyacrylamide gel electrophoresis, followed by membrane transfer and sealing with a rapid sealing solution for 15 min. Primary antibodies, including GAPDH (1:25000, 60004-1-lg), LIG1 (1:5000, 67840-1-lg), E-cadherin (1:5000, 20874-1-AP), N-cadherin (1:4000, 22018-1-AP), Snail (1:1000, 13009-1-AP), CCND1 (1:10000, 60186-1-lg), and CDK1 (1:5000, 19532-1-AP) were added and left to incubate overnight at 4°C on a shaking table. The next day, secondary antibodies were added and left to incubate at room temperature for 2 h. The membrane was then washed with tris-buffered saline-Tween (TBST), exposed to an enhanced chemiluminescence (ECL) developer (NCM Biotech, Suzhou, China), and imaged using a gel imager. The intensity of each band was quantified using ImageJ software.

### 2.17 EdU cell proliferation assay

The Cell-Light™ EdU kit (RiboBio, China) was used to evaluate cell proliferation, following the guidelines provided by the manufacturer. In brief, cells were exposed to EdU reagent for 2 h, followed by fixation with paraformaldehyde for 30 min, and neutralisation of excess aldehydes with glycine for 5 min. The cells were then stained with Apollo 567 and Hoechst 33342, each for a duration of 30 min. A fluorescence microscope was used to capture images of the cells, and the proliferation rate was quantified using ImageJ software.

### 2.18 Wound healing assay

T24 cells were cultured in a 6-well plate until reaching 80% confluence. A scratch was created by manually disrupting a small area of the monolayer using a pipette tip. Images of the scratch were taken immediately after the initial scratching and again after 24 h to monitor cell migration and wound closure. The images were analysed to determine the migration rate of the cells.

### 2.19 Transwell invasion assay

The cell invasion assay was conducted using 24-well transwell cell culture chambers (Corning, USA) with Matrigel (Corning, USA). Cells (8 × 10^4^) were placed in 150 μL of FBS-free medium in the upper chamber, while the lower chamber was filled with 600 μL of medium containing 10% FBS. After incubating for 24 h at 37°C and 5% CO_2_ (or 48 hours for the invasion assay), the cells on the lower surface of the chamber were fixed with 4% paraformaldehyde for 20 min and stained with crystal violet for 15 min. The invaded cells were counted in three randomly chosen fields for each chamber, and the experiments were performed in triplicate. Images were taken using a brightfield microscope (Olympus), and cell counting was performed using ImageJ software.

### 2.20 Cell apoptosis assay

Apoptosis was evaluated using an apoptosis kit from Elabscience (USA). For the apoptosis assay, 2 × 10^5^ cells were resuspended in 500 μL of 1× Annexin V Binding Buffer. Following this, 5 μL each of Annexin V-APC Reagent and Propidium Iodide (PI) Reagent were added to the cell suspension. After a gentle vortex mix, the mixture was left to incubate at room temperature in the dark for 15–20 min. Apoptosis was then analysed using a flow cytometer (Becton Dickinson, USA).

### 2.21 Cell cycle assay

Cell cycle analysis was performed by harvesting 1 × 10^6^ cells, which were then washed with phosphate-buffered saline (PBS) and fixed with 75% cold ethanol for 24 h at –20°C. The cells were then washed twice with PBS and stained with PI using the Cycletest Plus DNA Reagent Kit (BD Biosciences, USA) for 30 min at room temperature. Next, flow cytometry (Becton Dickinson, USA) was used to analyse the cells, and the distribution of the cell cycle was determined using the Cell Quest Modfit software.

### 2.22 CCK-8 assay

Cells were seeded in 96-well plates (Corning, Corning, NY, USA) at a density of 3 × 10^3^ cells per well and kept in a humidified chamber. To avoid evaporation of the medium, PBS was added around the plate. At specific time points, each well was supplemented with 100 µL of culture medium containing 10% CCK-8 (EnoGeneCell, Nanjing, China). After incubating for 2 h at 37°C, the optical density (OD) was measured for three consecutive days. Each sample was tested three times.

### 2.23 Data processing

Statistical analysis was performed using R (version 4.2.2) from the R Foundation for Statistical Computing, Vienna, Austria (https://www.R-project.org/), and GraphPad Prism software (version 9.5.0). Quantitative data are presented as mean ± standard deviation. The chi-square (χ2) test was used to examine the association between *LIG1* expression and the clinical pathological characteristics. Inter-group differences were analysed using Student’s t-test (unpaired, two-tailed) and one-way analysis of variance (ANOVA). The correlation between clinical features and *LIG1* expression levels was evaluated through logistic regression analysis. Survival differences between groups with high and low *LIG1* expression were assessed using Kaplan–Meier analysis and the log-rank test on the BEST website, based on cut-off points and overall survival. A p-value less than 0.05 was deemed statistically significant.

## 3 Results

### 3.1 DEGs and WGCNA

**Figure 1:**
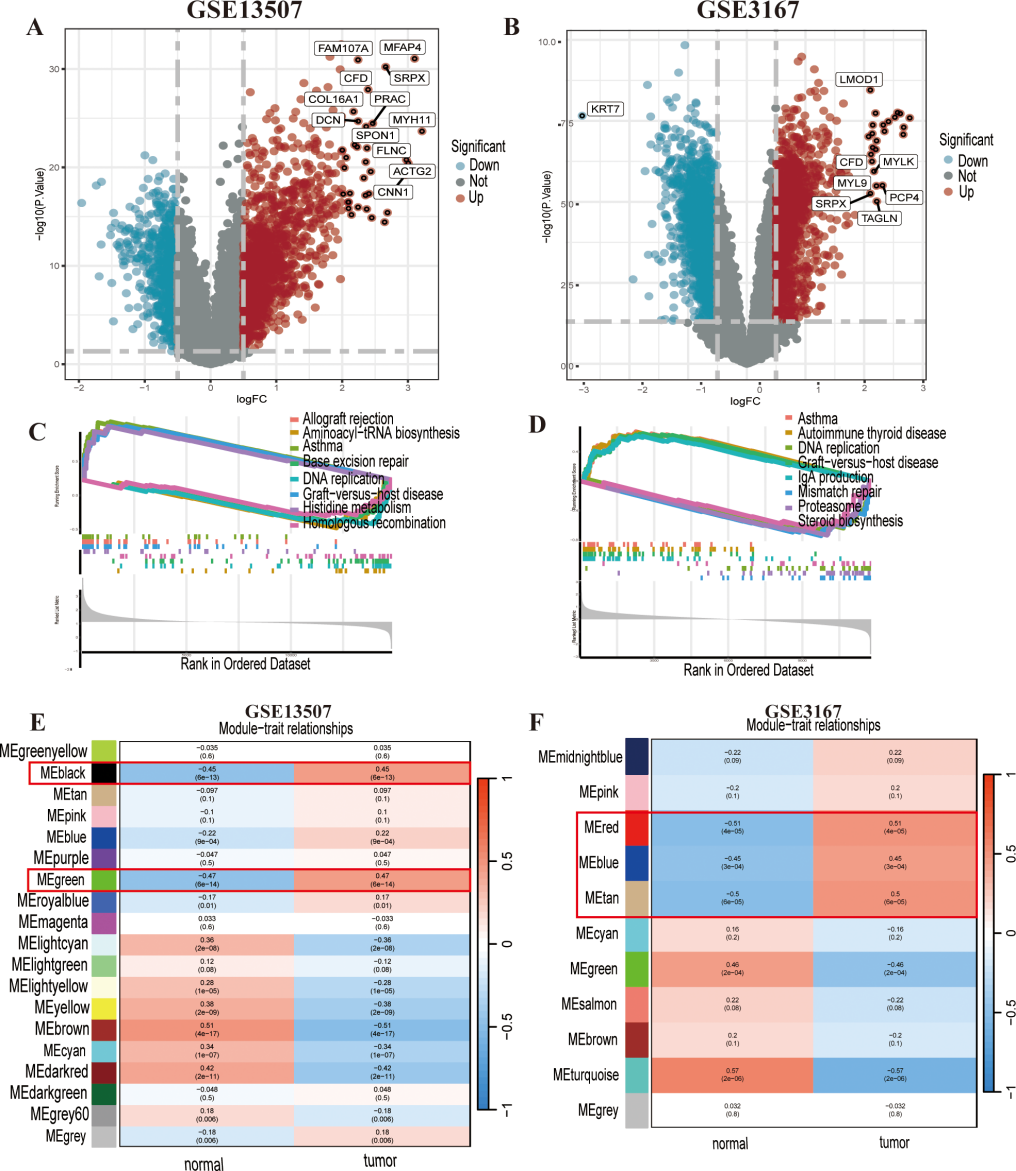
Differentially expressed genes (DEGs) and functional enrichment analysis of GSE13507 and GSE3167. **(A)** The DEGs in GSE13507 are shown in a volcano plot. **(B)** The DEGs in GSE3167 are shown in a volcano plot. **(C, D)** The gene set enrichment analysis (GSEA) suggested that GSE13507 and GSE3167 both showed enrichment for asthma, DNA replication, and graft-versus-host disease. **(E, F)** Weighted gene coexpression network analysis (WGCNA) of GSE13507 and GSE3167; the strongly positive correlation modules are indicated in the figure.

We conducted differential analysis of bladder urothelial carcinoma data based on cancer tissue and adjacent normal tissue.DEGs were identified from GSE13507 and GSE3167 (**Figures 1A, B**; **Supplementary Figures S1A, S1B**), which were subjected to Gene Set Enrichment Analysis (GSEA) to determine the diseases or functions regulated by the DEGs in the two datasets (**Figures 1C, D**). We found that asthma, graft-versus-host disease, and DNA repair were commonly enriched diseases or functions in both datasets, suggesting a potential association between bladder cancer development and changes in these functional genes. Furthermore, the WGCNA revealed that the black and green modules in GSE13507 (**Figure 1E**), and the blue, red, and tan modules in GSE3167 (**Figure 1F**), had the highest positive correlation with cancer.

### 3.2 PPI

**Figure 2:**
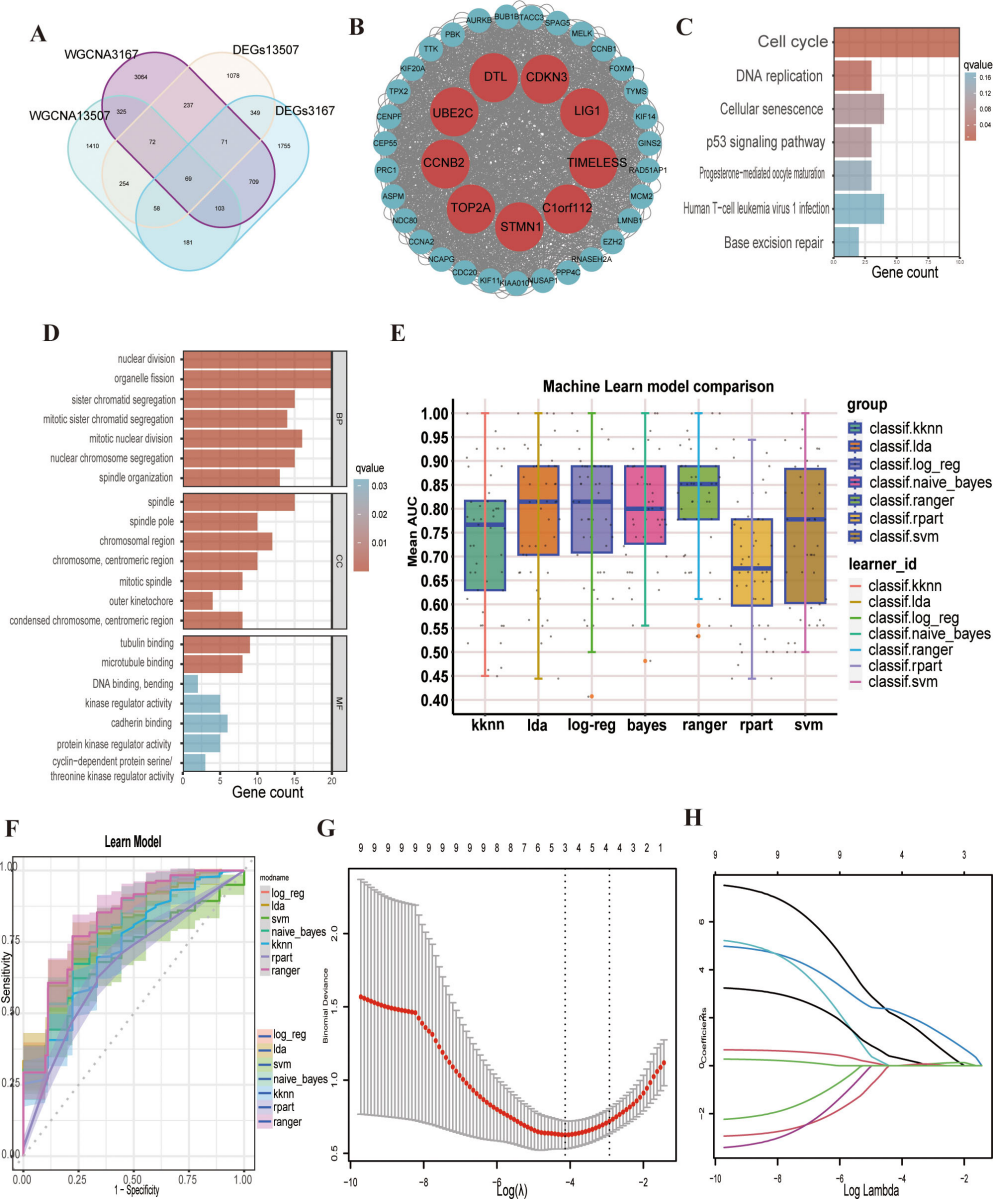
Screening and enrichment analysis of hub genes and related multiple machine learning. **(A)** The venn plot shows the intersected 69 genes. **(B)** Hub genes are shown in the protein–protein interaction (PPI) plot. **(C, D)** The enrichment analysis showed that nuclear division, organelle fission, sister chromatid segregation, mitotic sister chromatid segregation, mitotic nuclear division, spindle, tubulin binding, cell cycle, DNA replication, cellular senescence and the p53 signalling pathway were activated. **(E)** Machine learn model comparison of the nine hub genes. **(F)** Reciever operating characteristic (ROC) curve of the multiple machine learning models. **(G)** Binomial deviance of overall survival (OS) for the LASSO coefficient profiles. **(H)** LASSO coefficient profiles of genes.

The overlap between the identified modules and DEGs resulted in 69 genes showing the strongest positive correlation with cancer(**Figure 2A**). Subsequently,protein–protein interactions (PPIs) based on a network constructed from 69 intersecting genes identified nine key genes (LIG1, C1orf112, CCNB2, CDKN3, UBE2C, DTL, STMN1, TOP2A, and TIMELESS) as hub genes (**Figure 2B**).

### 3.3 Enrichment analysis

The KEGG pathway analysis results indicated that the hub genes were significantly enriched in pathways related to the cell cycle, DNA replication, cellular senescence, and the p53 signalling pathway (**Figure 2C**). GO enrichment analysis revealing that hub genes were predominantly involved in biological processes, such as nuclear division and organelle fission. In terms of cellular components, the genes were enriched in the spindle and chromosomal region, while at the molecular function level, they were associated with tubulin binding and microtubule binding(**Figure 2D**). Some current studies support our findings from the GO and KEGG enrichment analyses, reinforcing the reliability of our research.

### 3.4 Multi-machine learning

We subsequently used the hub genes from the PPI network to construct a predictive model. The GSE13507 dataset was employed as the training set, and the GSE3167 dataset was used for model validation. We applied seven distinct machine learning algorithms and optimised the parameters for each model through five iterations of ten-fold cross-validation (**Figure 2E**). The predictive accuracy of these models was evaluated by assessing the area under the curve (AUC) values in the validation set (**Figure 2F**). Following a thorough selection process, the “logreg” machine learning algorithm model, which had the highest AUC of 0.793, was selected (**Supplementary Figure S1C**). Additionally, a linear regression model was developed to assess the pathogenicity of the nine hub genes in bladder cancer. Using the LASSO regression algorithm, we pinpointed three key genes (*LIG1, STMN1* and *UBE2C*) that were strongly linked to the disease’s pathogenicity among the hub genes (**Figures 2G, H**). *LIG1* had the highest weight in the LASSO regression model, warranting a more in-depth analysis of *LIG1*.

### 3.5 Survival analysis

**Figure 3:**
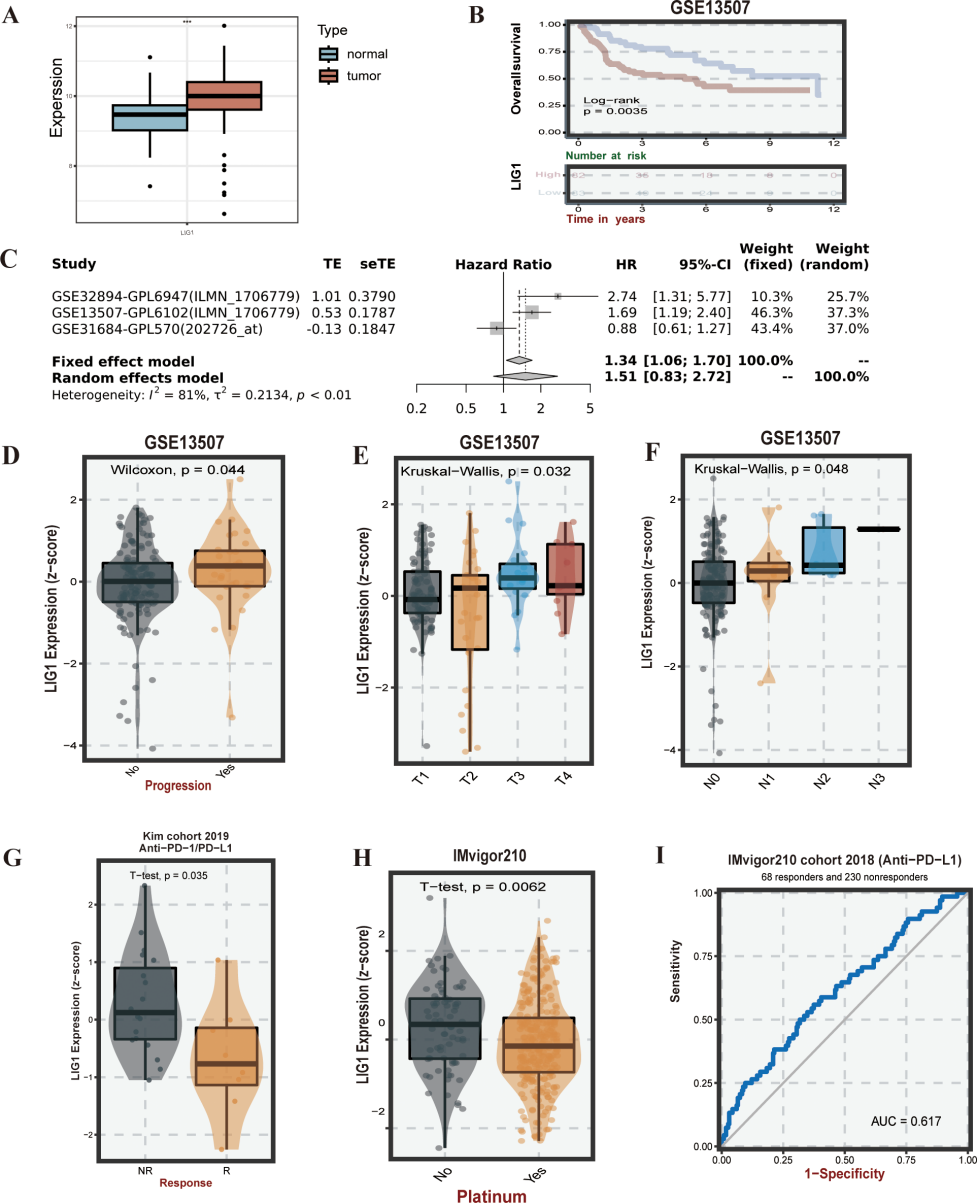
*LIG1* expression, survival, and drug susceptibility analysis. **(A)** Expression of *LIG1* in normal tissues and bladder cancer tissues (human bladder cancer). **(B)** Overall survival analysis of *LIG1* expression. **(C)** The meta-survival analysis of *LIG1* expression (results from GENT2). **(D–F)** *LIG1* expression and bladder cancer progression correlation analysis (stage T and stage N). **(G–I)** Correlation analysis of *LIG1* expression and immunotherapy. ***P value < 0.001.

Based on the transcriptomic data from GSE13507 and matched clinical data, we found that *LIG1* expression in bladder cancer was higher than that in normal tissues (**Figure 3A**), and patients with high *LIG1* expression had shorter survival times (**Figure 3B**). In the meta-survival analysis results, the hazard ratio (HR) was 1.34 for the fixed effects model and 1.51 for the random effects model, further confirming that *LIG1* is a significant adverse prognostic factor in bladder cancer (**Figure 3C**). *LIG1* also plays a crucial role in the progression of bladder cancer; higher expression of *LIG1* was associated with an increased likelihood of cancer progression (**Figure 3D**). According to the clinical data from GSE13507, the expression of *LIG1* correspondingly increased with higher T stage bladder cancer (**Figure 3E**), indicating enhanced proliferative capacity in bladder cancer with high *LIG1* expression. Similarly, the expression of *LIG1* increased with higher N stage bladder cancer (**Figure 3F**), suggesting that bladder cancer with high *LIG1* expression is more prone to lymphatic invasion of the surrounding tissues.

### 3.6 Immune inflation

Based on pre-processing using the BEST online platform, we conducted immune infiltration analysis of bladder cancer with high *LIG1* expression using algorithms such as CIBERSORT, CIBERSORT_ABS, EPIC, ESTIMATE, MCPcounter, Quantiseq, TIMER, xCell, etc., on the following datasets: GSE154261, GSE39281, GSE52219, GSE70691, GSE37815, GSE48276, GSE69795, GSE19423, GSE48075, IMvigor210, GSE13507, TCGABLCA and GSE31684. In the Quantiseq algorithm, the infiltration of Tregs, M1 macrophages and CD8+T cells was prominent. In the CIBERSORT algorithm, the infiltration of follicular helper T (Tfh) cells, M1 macrophages, M0 macrophages, naive B cells and CD8+T cells was notable. The TIMER algorithm indicated higher infiltration levels of B cells. The MCPcounter algorithm showed significant infiltration levels of CD8+T cells and B lineage. In the CIBERSORT_ABS algorithm, Tfh cells, M0 macrophages and naive B cells exhibited higher infiltration levels. The xCell algorithm revealed higher counts of Th1 cells, gamma delta T (Tgd) cells, megakaryocytic-erythroid progenitors (MEPs), epithelial cells, osteoblasts and Th2 cells. In the EPIC algorithm, endothelial and natural killer (NK) cells showed higher count levels (**Supplementary Figure S2**).

### 3.7 Immune response

**Figure 4:**
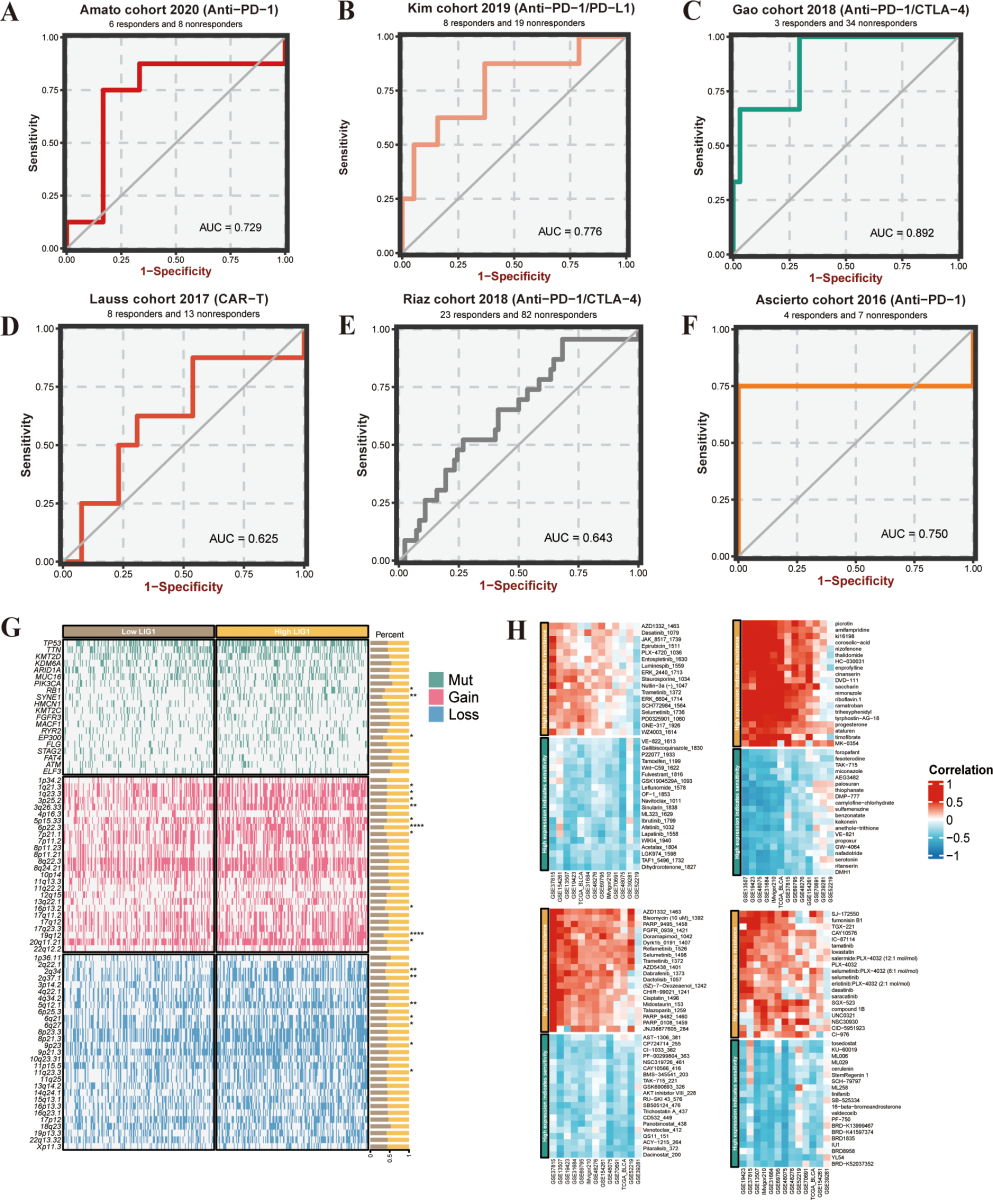
Immunotherapy prediction and analysis of somatic mutations and copy number variance (CNV). **(A–F)** Immunotherapy sensitivity prediction of the different cohorts. **(G)** Waterfall plot of somatic mutations and CNV between low *LIG1* expression and high *LIG1* expression groups. **(H)** Heatmap of *LIG1* candidate drug predictions. *P < 0.05; **P < 0.01; ***P < 0.001.

Based on the KIM cohort, we found that high expression of the *LIG1* gene resulted in ineffectiveness of anti-PD-1 and anti-PD-L1 immunotherapy (**Figures 3G**, **4B**). In the cohort, the low *LIG1* expression group showed a better immune response, while the high expression group exhibited a poorer immune response. Furthermore, based on the data from the IMvigor210 cohort, we observed that the high *LIG1* expression group had poorer treatment outcomes with platinum-based chemotherapy drugs and a lower response to anti-PD-L1 therapy (**Figures 3H, I**). Subsequently, we used the Amato cohort, GAO cohort, Lauss cohort, Riaz cohort and Ascierto cohort to plot the AUC values, confirming that the high *LIG1* expression group had poor responsiveness to anti-PD-1, anti-CTLA-4, anti-PD-L1 and CAR-T immunotherapy (**Figures 4A-F**).

### 3.8 Genomic alteration analysis

In our investigation of the association between *LIG1* expression levels and specific genomic features of bladder cancer, we performed somatic mutation and CNV analyses using the TCGA bladder cancer database using the BEST online platform. The analysis identified the top 20 mutated genes, with notable mutations in *RB1, SYNE1* and *EP300* observed in the comparison between the high and low *LIG1* expression groups. Notably, the high *LIG1* group exhibited amplifications in chromosomal segments 1q21.3, 1q23.3, 3p25.2, 3q26.33, 5p15.33, 6p22.3, 7p21.1, 16p13.2, 19q12 and 20q11.21, along with deletions in chromosomal segments 2q34, 2q37.1, 5q12.1, 6q21, 6q27, 9p23 and 11q23.3 (**Figure 4G**).

### 3.9 Immune candidate drugs

In the drug treatment prediction analysis, the GDSC1 database analysis was used to show that bladder cancer with high *LIG1* expression exhibited resistance to a series of drugs including AZD1332, PARP9495, Dyrk1b0191, AZD5438, Bleomycin1392, Bleomycin1378, Refametinib1526, Selumetinib1498 and others. In the GDSC2 database analysis, high *LIG1* expression in bladder cancer showed resistance to drugs such as ERK2440, Staurosporine, Nutlin-3a, Trametinib, ERK6604, Selumetinib, SCH772984, AZD1332, Dasatinib1079, Luminespib1669 and more. In the CTRP database, we observed that bladder cancer with high *LIG1* expression was resistant to drugs including SGX-523, compound 1B, NSC30930, SJ-172550, Fumonisin B1, TGX-221, CAY10576, Lovastatin, Selumetinib, CID-5951923 and others. In the PRISM database, we found that the high LIG1 expression group exhibited resistance to drugs such as Saccharin, Trihexyphenidyl, Nimorazole, Ramatroban, Enprofylline, Corosolic-acid, Cinansern, Thalidomide, HC-030031, Timofibrate and more. These data indicate that high *LIG1* expression is indeed a risk factor for bladder cancer drug treatment (**Figure 4H**).

### 3.10 Identifying the clusters

**Figure 5:**
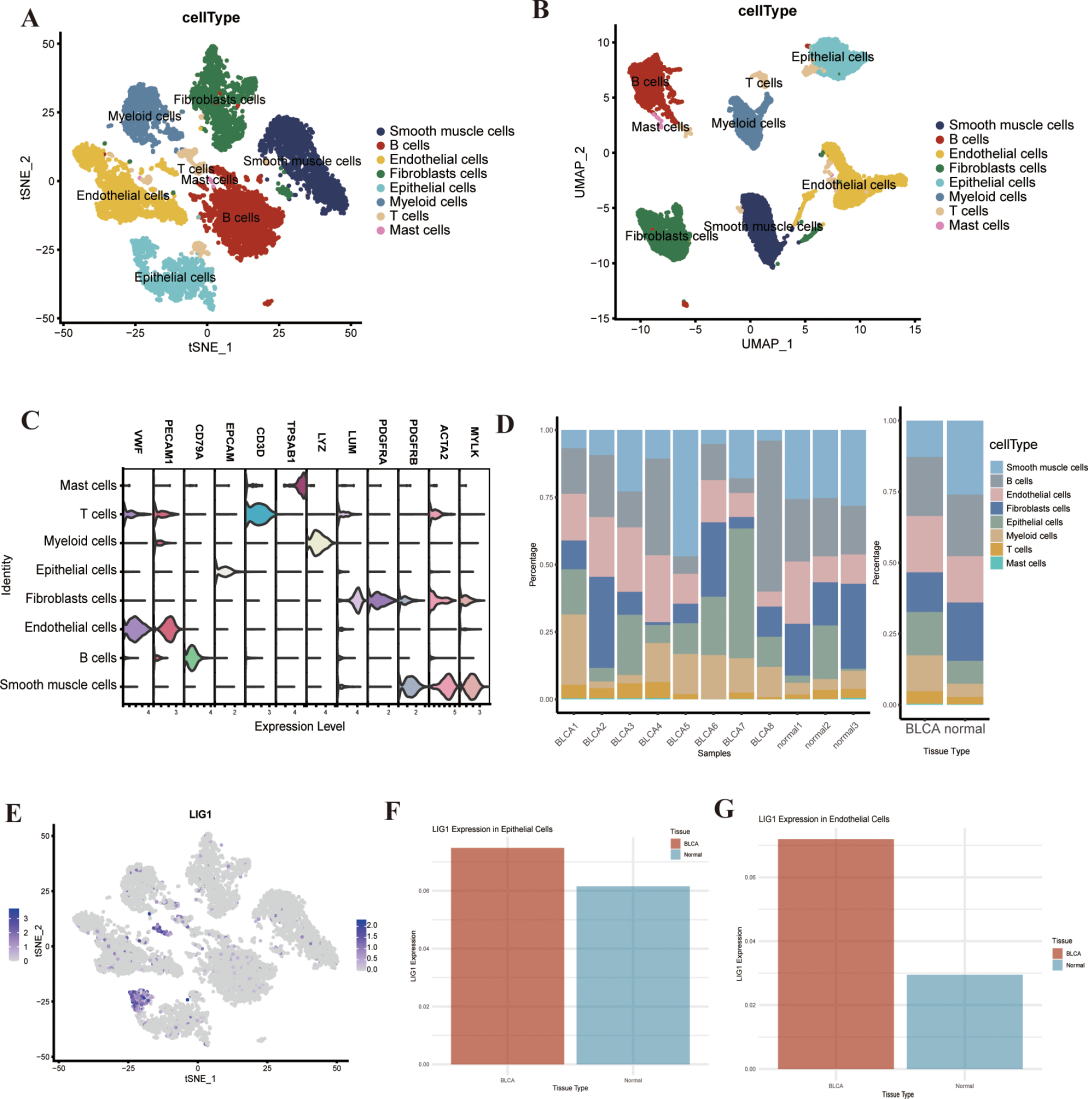
**(A, B)** tSNE or UMAP plots to identify each cell type in bladder cancer. **(C)** The violin plot shows the cell markers to identify each cell type. **(D)** The percentage plot of different types of cells in bladder cancer and adjacent tissues. **(E)** *LIG1* expression in the different types of cells. **(F)** The expression of *LIG1* in epithelial cells. **(G)** The expression of *LIG1* in endothelial cells.

In the tSNE dimensionality reduction clustering, we derived 34 clusters (**Supplementary Figure S3A**) and determined the expression levels of the features for each cluster (**Supplementary Figures S3B, S3C**). This analysis led to the identification of eight clusters, including mast cells (TPSAB1+), T cells (CD3D+), myeloid cells (LYZ+), epithelial cells (EPCAM+), fibroblasts (LUM+), endothelial cells (VWF+), B cells (CD79A+) and smooth muscle cells (ACTA2+) (**Figures 5A-C**). Utilising the “Findallmarkers “function, we ascertained that the *LIG1* gene was primarily distributed in clusters “17” and “21”, specifically in the endothelial cells and epithelial cells clusters (**Figure 5E**). Consequently, we investigated the relationship between tissue expression levels in the normal and tumour groups, discovering a significant increase in myeloid cells, T cells, epithelial cells and endothelial cells in the tumour group, which is likely associated with the previously identified *LIG1* gene (**Figure 5D**). Upon exploring expression levels, we observed that *LIG1* expression was significantly elevated in epithelial cells and endothelial cells, leading us to hypothesise that *LIG1* may play a role in tumour proliferation (**Figures 5F, G**).

### 3.11 Cellchat

**Figure 6:**
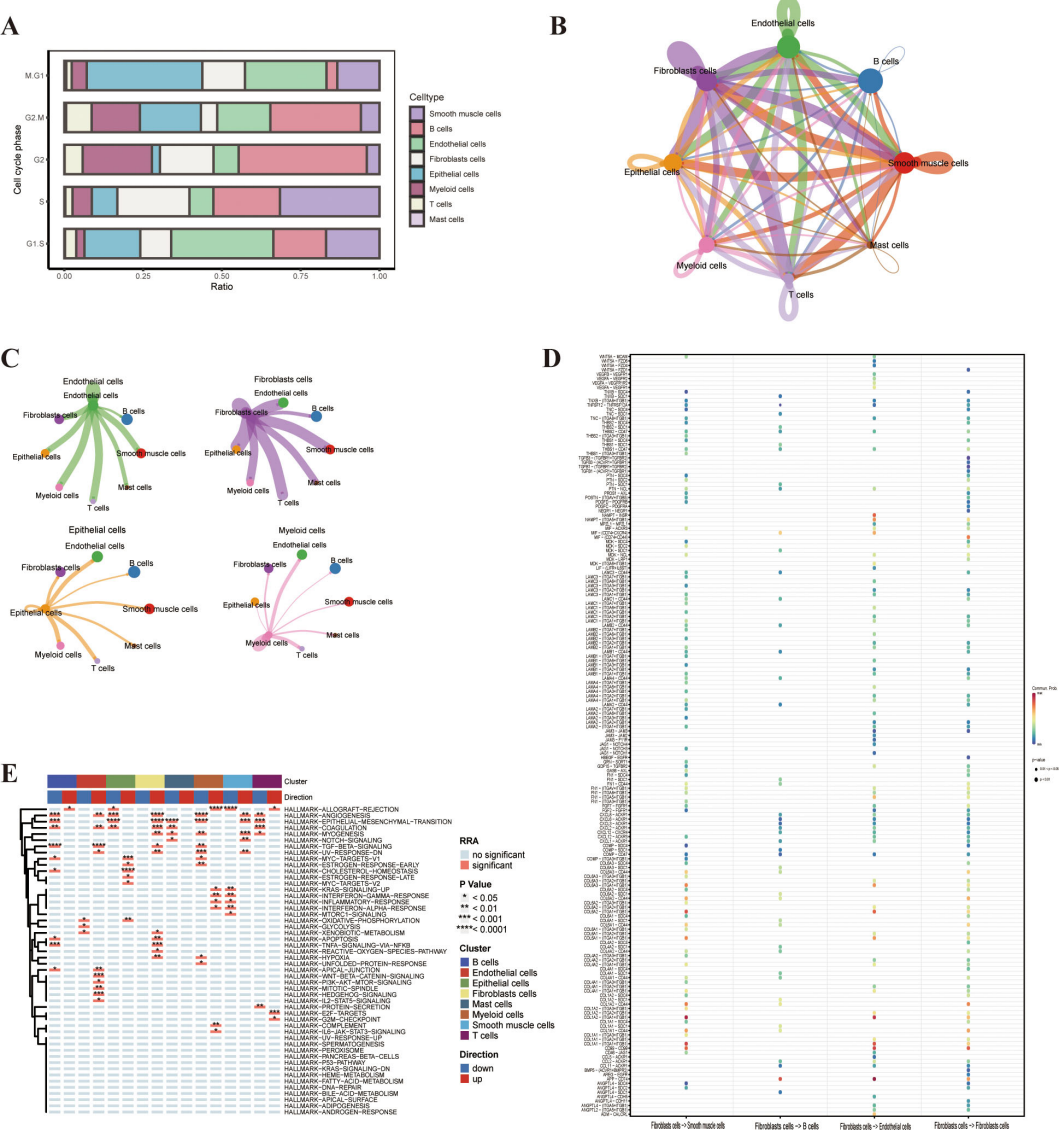
Cell cycle phase, cellchat, and gene set variation analysis (GSVA). **(A)** Cell cycle phase of the different cell types. **(B)** Cellchat analysis of the different cell types in bladder cancer. **(C, D)** Interaction of the different cell types in bladder cancer. **(E)** GSVA enrichment analysis of the different cell types.

Drawing on the findings from the GO and KEGG enrichment analysis, we assessed the cell cycle-related data through an analysis of the single-cell sequencing data. This revealed an increase in the proliferation of epithelial cells, endothelial cells and T cells,aligning with the function of the key genes we previously identified(**Figure 6A**). To delve deeper into the interactions among the eight cell clusters, we employed the “cellchat” tool for cell interaction analysis.We discovered that fibroblasts had robust interactions with epithelial cells, endothelial cells, T cells and smooth muscle cells, suggesting that these cell interactions could influence fibroblast generation and the immune microenvironment(**Figures 6B, C**). Specifically, smooth muscle cells and fibroblasts demonstrated strong reciprocal interactions via the COL1A2-(ITGA1+ITGB1) and COL1A1-(ITGA1+ITGB1) pathways. Endothelial cells and fibroblasts exhibited strong interactions through the APP-CD74 pathway, with CD74 being a marker of macrophages, implying that endothelial fibrosis might be mediated through the activity of macrophages (**Figure 6D**).

### 3.12 Gene set variation analysis

To investigate the potential impact of the *LIG1* gene on cluster functions, we conducted GSVA on the eight clusters of single-cell sequencing data. The DEGs in the endothelial cells cluster were mainly enriched in pathways such as TGF-β signalling, WNT-β-CATENIN signalling and HEDGEHOG signalling. The DEGs in the epithelial cells cluster were mainly enriched in functions related to ANGIOGENESIS, EPITHELIAL–MESENCHYMAL TRANSITION, MYC-TARGETS-V1 and CHOLESTEROL HOMEOSTASIS. The DEGs in the fibroblast cluster were mainly enriched in functions related to ANGIOGENESIS and EPITHELIAL–MESENCHYMAL TRANSITION, while the DEGs in the smooth muscle cells cluster were mainly enriched in functions related to EPITHELIAL–MESENCHYMAL TRANSITION and MYOGENESIS (**Figure 6E**). Therefore, we hypothesise that the proliferation of endothelial cells leading to epithelial–mesenchymal transition (EMT) may be due to the action of CD74.

### 3.13 Bladder cancer exhibits an upregulation of *LIG1*

**Figure 7:**
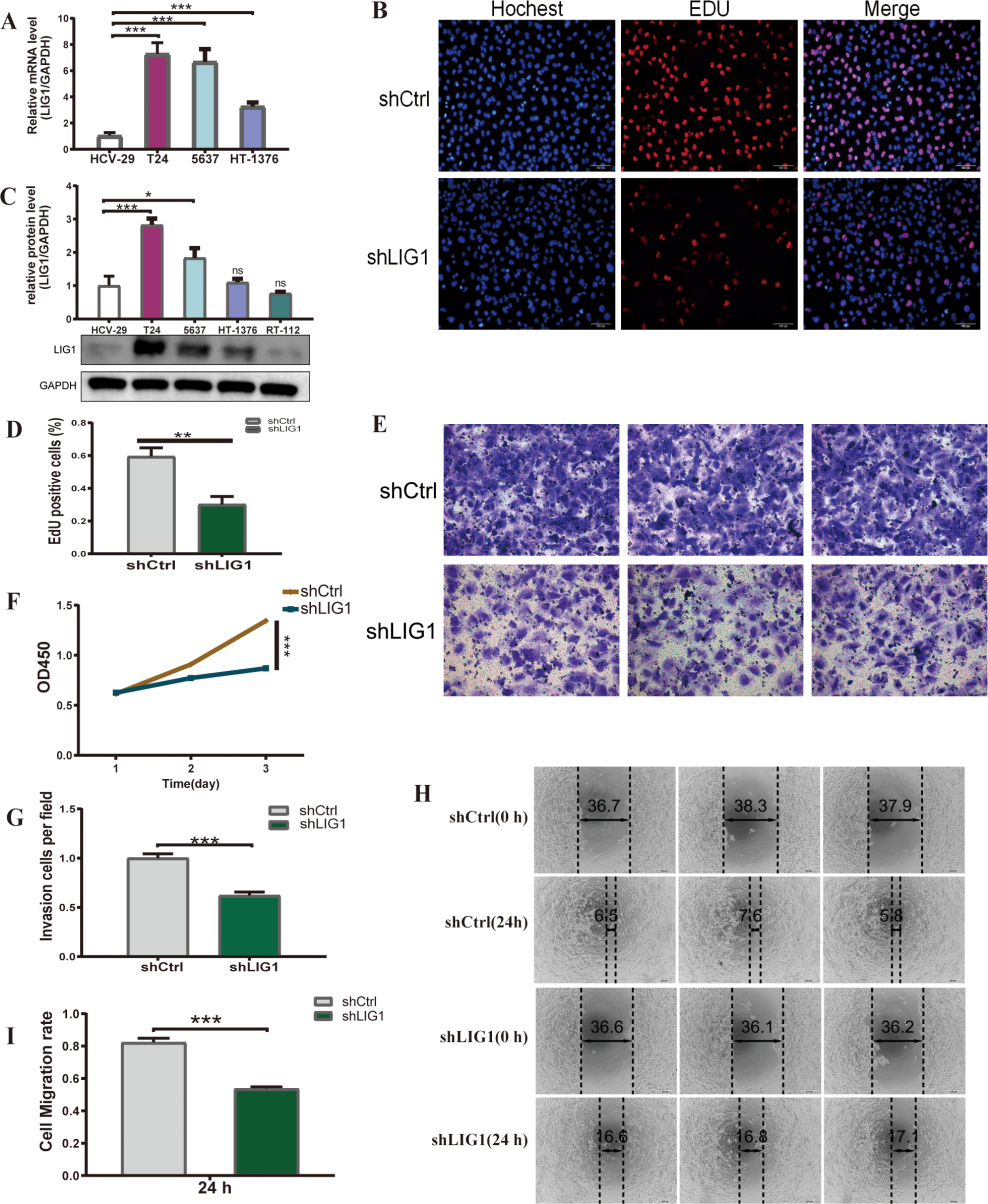
*LIG1* is involved in the proliferation and migration of bladder cancer cells. **(A)** The qRT-PCR results show the mRNA expression level of *LIG1* in bladder cancer cells (T24, 5637, HT-1376) and normal bladder epithelial cells (HCV-29). **(B, D)** The cell proliferation capacity was detected using the EdU assay. **(C)** The expression level of LIG1 protein in bladder cancer cells (T24, 5637, HT-1376, RT-112) and the normal bladder epithelial cell line (HCV-29) was measured by Western blotting. **(F)** Cell viability was measured using the CCK-8 assay. **(E, G)** The effect of *LIG1* knockdown on T24 cell invasion was evaluated using the transwell assay. **(H, I)** Quantitative statistics of transwell migration assays in T24 cells. A wound healing assay was performed to investigate the effects of *LIG1* knockdown on the migration on T24 cells (*p < 0.05, **p < 0.01, ***p < 0.001, ns, not significant).

In the GSE13507 cohort, *LIG1* expression was markedly elevated in bladder cancer tumour samples compared to normal samples.This increase was also observed in T24, 5637 and HT-1376 cells compared to HCV-29 cells, as evidenced by qRT-PCR analysis (**Figure 7A**). Subsequently, we performed Western blotting to evaluate the protein levels, which showed a significant increase in LIG1 protein expression in T24 and 5637 cells compared to HCV-29 cells (**Figure 7C**). Given the collective data from qRT-PCR and Western blotting, we chose T24 cells for additional experiments. We then established lentivirus-mediated *LIG1* knockdown in T24 cells and carried out a series of cellular experiments to explore the effect of *LIG1* on bladder cancer cell phenotypes.

### 3.14 *LIG1* knockdown decreases the growth of bladder cancer cells

We established lentivirus-mediated *LIG1* knockdown in T24 cells and subsequently conducted a range of cellular experiments to examine the influence of *LIG1* on bladder cancer cell phenotypes. The EdU assay results demonstrated a reduction in the number of bladder cancer cells in the *shLIG1* group compared to the *shCtrl* group (**Figures 7B, D**). Furthermore, the CCK-8 assay corroborated these results, suggesting that *LIG1* knockdown notably decreased bladder cancer cell proliferation (**Figure 7F**).

### 3.15 *LIG1* knockdown suppresses the migratory and invasive abilities of bladder cancer cells

We evaluated the influence of *LIG1* on the migration ability of bladder cancer cells, a key aspect in tumour formation, using wound healing assays. The findings showed a notable decrease in cell migration in the *shLIG1* group compared to the *shCtrl* group (**Figures 7H, I**). The transwell assay yielded similar results, suggesting that *LIG1* has a substantial role in the migration ability of bladder cancer cells (**Figures 7E, G**).

### 3.16 *LIG1* knockdown enhances bladder cancer cell apoptosis

**Figure 8:**
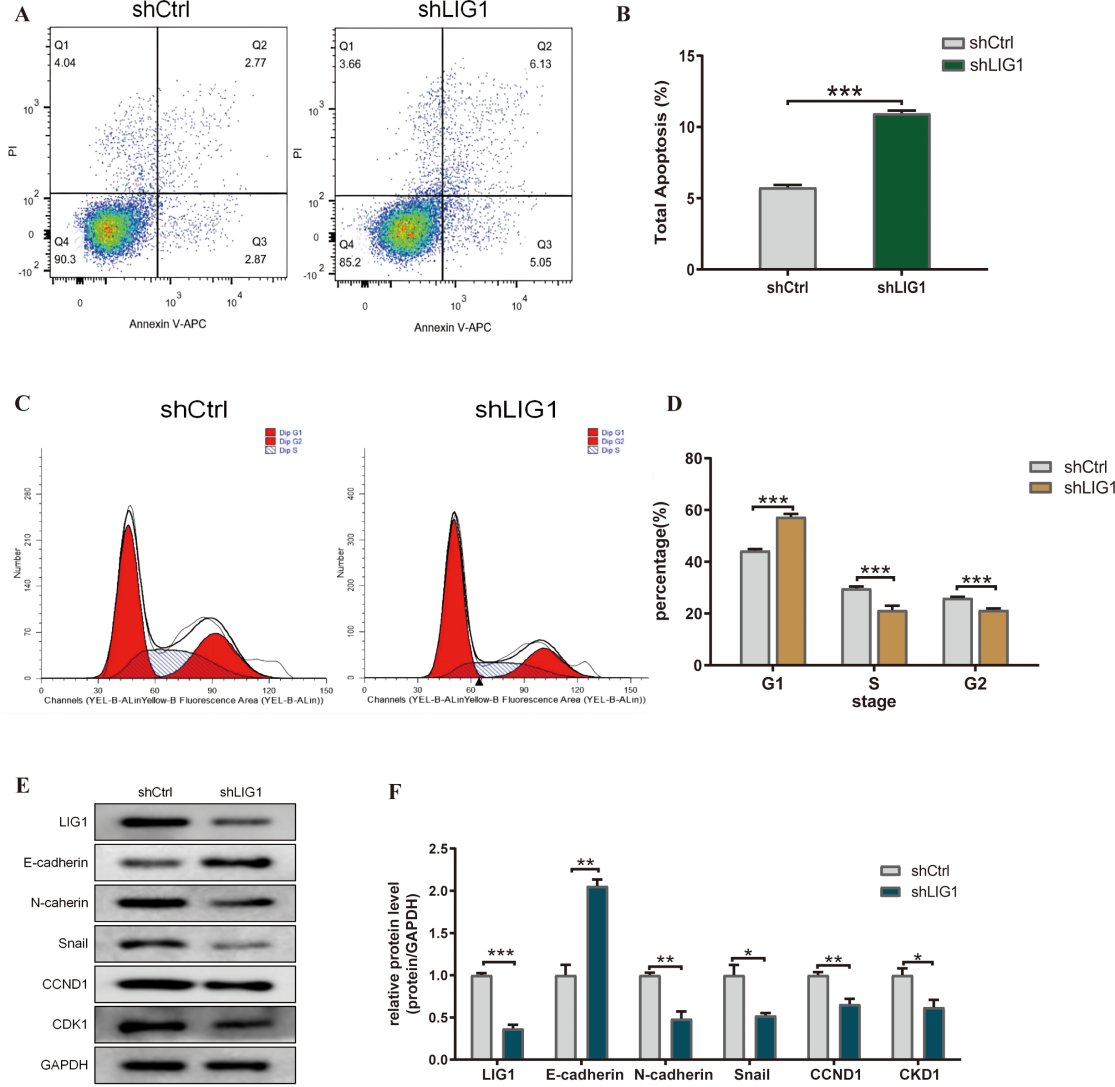
*LIG*1 knockdown affects the cell cycle, apoptosis and epithelial–mesenchymal transition (EMT) of bladder cancer cells. **(A, B)** The effect of *LIG1* knockdown on apoptosis of T24 cells was analysed by flow cytometry with Annexin V-APC/PI staining. **(C, D)** Flow cytometry showed that *LIG1* silencing delayed the cell cycle of T24 cells. **(E, F)** In T24 cells, motor inhibition caused by *LIG1* silencing was associated with EMT inhibition. EMT markers (E-cadherin, N-cadherin, Snail, CCND1 and CKD1) were detected by Western blotting. (*p < 0.05, **p < 0.01, ***p < 0.001, ns, not significant).

Our research examined the effect of *LIG1* on the apoptosis of bladder cancer cells, a vital characteristic of tumour cell activity, using flow cytometry. The data indicated that knockdown of *LIG1* resulted in a marked increase in the apoptosis rate of bladder cancer cells, in both the early and late stages, as well as in general (**Figures 8A, B**).

### 3.17 *LIG1* knockdown inhibits the bladder cancer cell cycle

From the KEGG enrichment analysis, we noticed a significant link with the cell cycle. Consequently, we assessed whether *LIG1* influenced the cell cycle of bladder cancer cells. Our findings indicated that knockdown of *LIG1* resulted in cell division being predominantly halted in the G1 phase (**Figures 8C, D**).

### 3.18 *LIG1* knockdown inhibits EMT in bladder cancer cells

Due to the results of the cell communication and GSVA enrichment analysis, we concluded that *LIG1* may induce fibrosis through the proliferation of endothelial cells and epithelial cells; therefore, *LIG1* may regulate EMT in bladder cancer. The Western blotting results showed that knockdown of *LIG1* inhibited EMT in bladder cancer cells (**Figures 8E, F**).

## 4 Discussion

The mortality rate of bladder cancer ranks 13th worldwide (1). In developing countries, the mortality rate of bladder cancer in men is still increasing (15). Despite significant progress in surgery, radiotherapy, chemotherapy and immunotherapy, the overall treatment effect has not significantly improved. Furthermore, the recurrence rate of bladder cancer is high, the life expectancy of muscle invasive bladder cancer is relatively short and the heterogeneity of bladder cancer patients is strong (16–18). Therefore, there is an urgent need to find new therapeutic targets that may help us improve the therapeutic treatment of bladder cancer.

In our study, we obtained 69 genes by intersecting the DEGs in the GEO13507 and GEO3167 datasets with the WGCNA analysis (19) of the normal and cancer groups. These 69 genes identified nine hub genes through the PPI network analysis. Finally, three key genes were screened through machine learning. At present, it is very common to use various forms of genetic information to study the prognosis of bladder cancer (20). A common pattern of this kind of research is to select gene sets and model them through LASSO and other algorithms. These models provide some assistance and reference for clinical practice; however, the drawback of this approach is that a specific set of genes may initially exclude the vast majority of genetic information. On the other hand, specific modelling methods are often not conducive to achieving the highest predictive ability of the model. Thanks to the rapid progress of machine learning, the simultaneous use of multiple machine learning algorithms can improve the predictive ability of the model to a certain extent (21).

The three key genes identified were *STMN1, UBE2C*, and *LIG1*. In previous studies, Xuan Zhu and others found that circST6GALNAC6 acts as a sponge, directly binding to miR-200a-3p to regulate the expression of stathmin (STMN1). In addition, STMN1 is involved in the circST6GALNAC6/miR-200a-3p axis-regulated BCa-EMT and metastasis (22). Ubiquitin-conjugating enzyme 2C (UBE2C) is involved in many cellular processes and the tumour progression of various cancers. In the study by Bor-Hwang Kang, UBE2C was reported to be a potential biomarker for the occurrence and prognosis of tongue squamous cell carcinoma (23).

We chose *LIGI* for more focused research. To our knowledge, there is currently no research related to *LIG1* and bladder cancer. LIG1 is one of the four DNA ligases in mammalian cells, and its gene is located at 19q13.2-13.3, a region often deleted in various types of tumour cells. As a broad-spectrum DNA repair gene, it participates in the ligation of Okazaki fragments in the lagging strand of double-stranded DNA synthesis and is involved in DNA excision repair (24). Early studies have shown that a *LIG1* deficiency can lead to Bloom syndrome, characterised by a higher frequency of chromosomal breaks and rearrangements, more frequent sister chromatid exchanges and slowed DNA replication, which can result in a higher incidence of tumours in the immune system of patients (25). In research on ovarian cancer, overexpression of *LIG1* and *LIG3* is associated with aggressive phenotypes, platinum resistance and lower progression-free survival (PFS) (26, 27). Smoking is currently recognised as the most common environmental exposure factor for lung cancer. The smoke from burning tobacco contains a large amount of polycyclic aromatic hydrocarbons, most of which can form adduct molecules after being activated in the human body and covalently binding to DNA, thereby inducing mutations (28). However, approximately 85% of smokers do not develop lung cancer (29), which may be due to differences in lung cancer susceptibility among individuals, including variations in DNA repair capabilities (30). As a DNA ligase gene, in the DNA excision repair process, after going through steps such as recognising the incorrect site, excising the wrong fragment and synthesising the correct fragment, the ligase encoded by *LIG1* is required to connect the new fragment to the DNA strand, completing the repair. Therefore, the function of LIG1, to some extent, determines the efficiency of the entire DNA excision repair system. In lung cancer research, it is believed that a polymorphism in exon 6 of the DNA ligase gene *LIG1* may be associated with susceptibility to lung adenocarcinoma and squamous cell carcinoma. A meta-analysis on the relationship between *LIG1* gene polymorphisms and lung cancer risk suggested that the rs156641 polymorphism was significantly associated with lung cancer risk (31). It is well known that smoking is the most significant risk factor for urothelial cell carcinoma (32). We have, for the first time, found a correlation between the *LIG1* gene and the proliferation and invasiveness of urothelial tumour cells, with extensive validation performed through cell experiments.

Additionally, we analysed the distribution of these genes in cell populations through single-cell sequencing data. Surprisingly, we found that most of the genes screened out by machine learning were highly expressed in epithelial cells. This is significant because urothelial carcinoma originates from epithelial cells. There is also a noticeable difference in the expression of *LIG1* in epithelial cells between the healthy and tumour groups. For the first time in bladder cancer cells, we analysed the function of the *LIG1* gene. After inhibiting its expression in cells with *shLIG1*, functional experiments revealed a reduction in cell proliferation, migration and invasiveness. Furthermore, cell apoptosis increased, and there was a rise in the G1 phase of the cell cycle with a decrease in the S/G2 phases, leading to reduced cell division. Additionally, KEGG analysis suggested that *LIG1* might affect tumour proliferation and invasiveness through the EMT pathway. Western blot experiments showed that after LIG1 expression was inhibited by the shRNA, the expression of E-cadherin significantly increased, while the expression of N-cadherin, Snail, CCND1, and CDK1 markedly decreased. This suggests that LIG1 can regulate the EMT pathway. Type 3 EMT is usually associated with tumour progression, especially the progression from non-muscular invasive bladder cancer to muscular invasive bladder cancer (33). However, our current understanding of the LIG1 signalling pathway is still not extensive enough, and further research is needed to elucidate it.

In summary, through the combination of machine learning and single-cell sequencing analysis, we have identified a new prognostic gene for urothelial carcinoma, *LIG1*, which may be related to the proliferation and invasiveness of urothelial carcinoma. It is possible that it exerts its effects by regulating the EMT signalling pathway. Based on the results of previous studies on the *LIG1* gene, further in-depth research is currently needed. *LIG1* has the potential to be a novel therapeutic target for urothelial carcinomas and could help us to better understand the relationship between smoking, a well known risk factor, and urothelial carcinoma susceptibility.

Our study has some limitations. First, the number of patients in our GEO database is relatively small. Second, in our experiments, we have not conducted an in-depth study on the signalling pathway of LIG1, nor have we validated our findings in animal experiments. Finally, the expression of LIG1 in urothelial carcinoma has not been studied. These shortcomings are what we will continue to investigate in our next steps. More prospective and basic research is needed to elucidate the details.

## Data Availability

The original contributions presented in the study are included in the article/**Supplementary Material**. Further inquiries can be directed to the corresponding authors.
