# Supplementary figures and images for: LIG1 is a novel marker for bladder cancer prognosis: evidence based on experimental studies, machine learning and single-cell sequencing
Source: Front Immunol. 2024 Aug 21;15:1419126. doi: 10.3389/fimmu.2024.1419126 (PMC11371609; doi:10.3389/fimmu.2024.1419126)

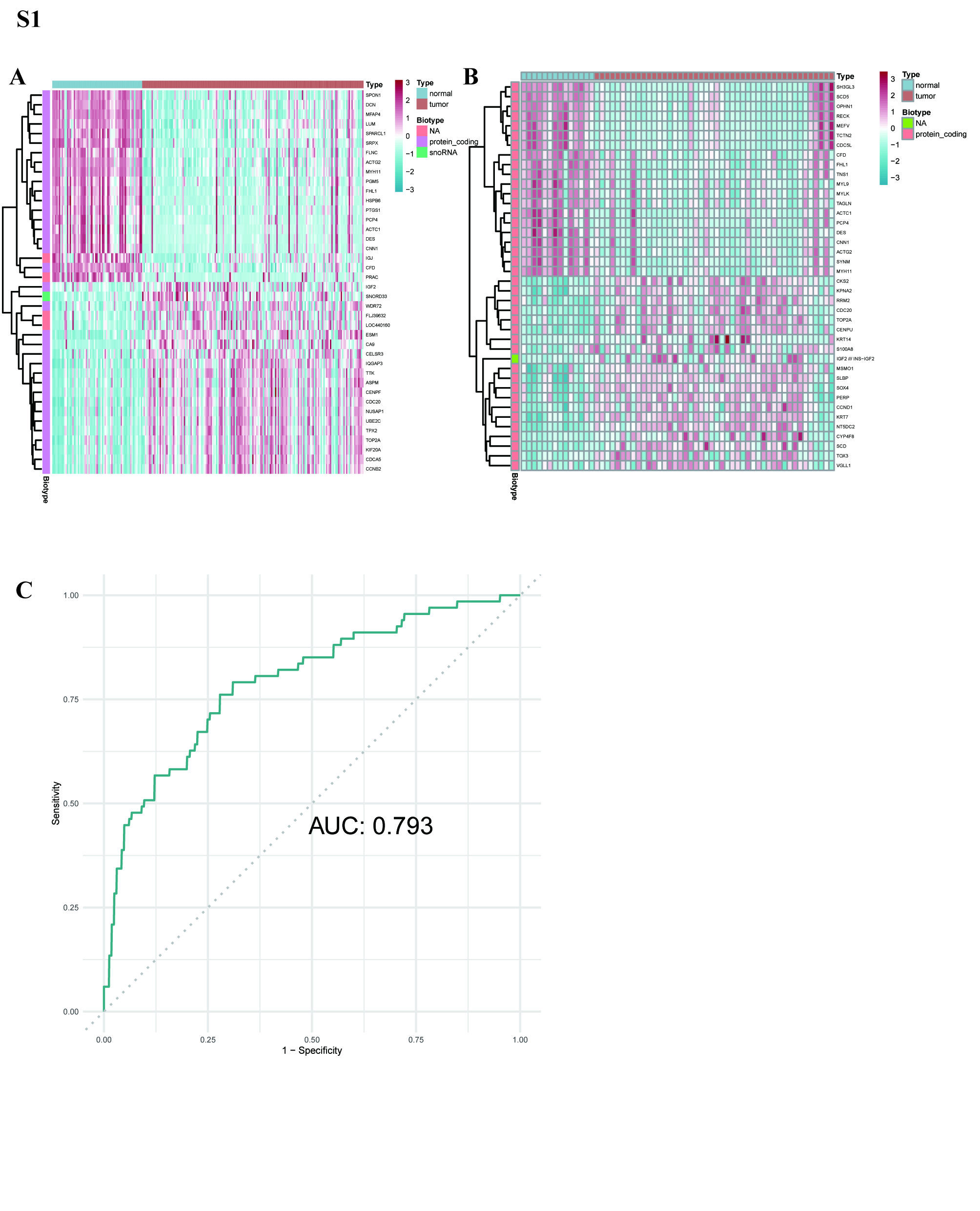

Supplement: Supplementary file 1 [file Image1.tif]

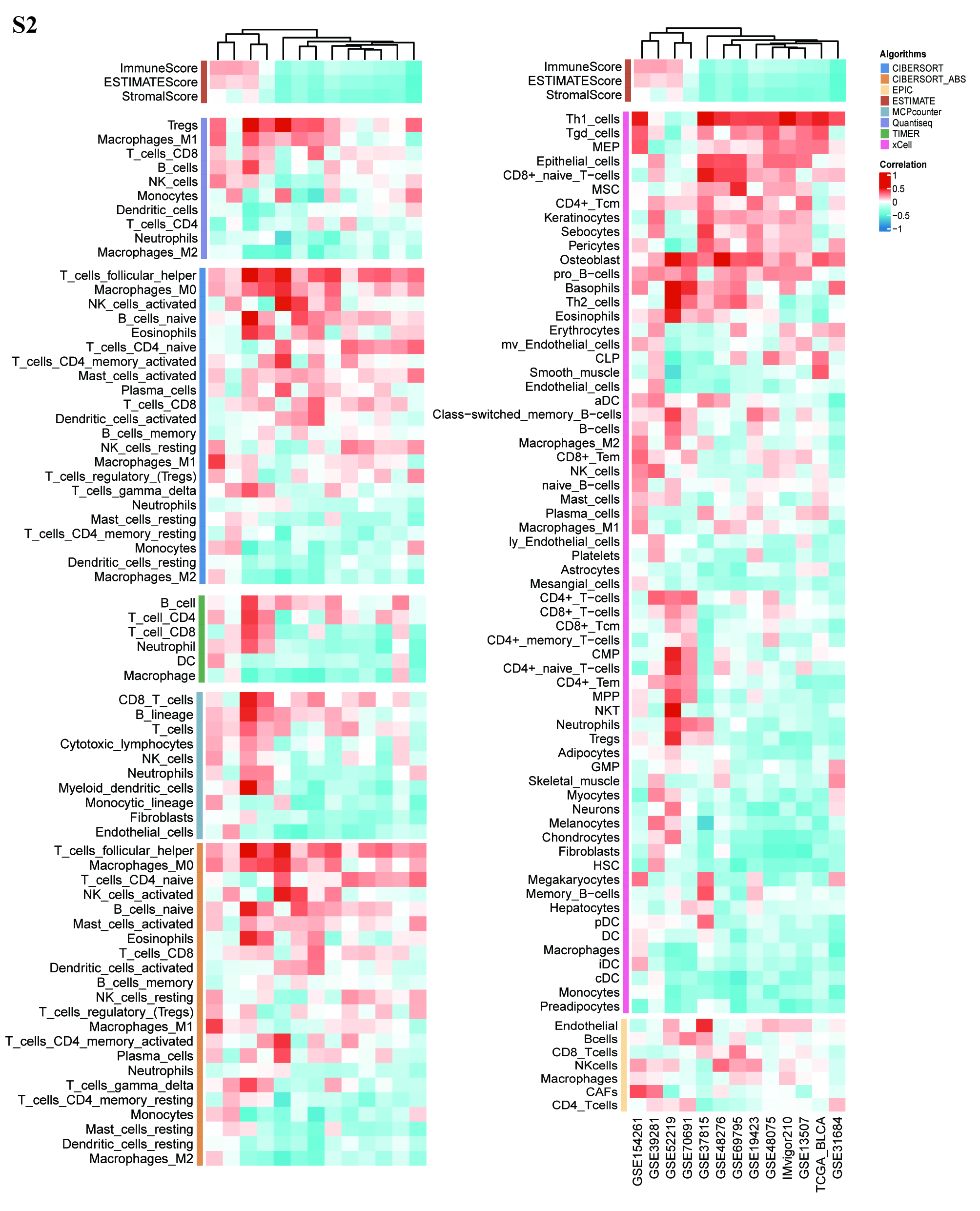

Supplement: Supplementary file 2 [file Image2.tif]

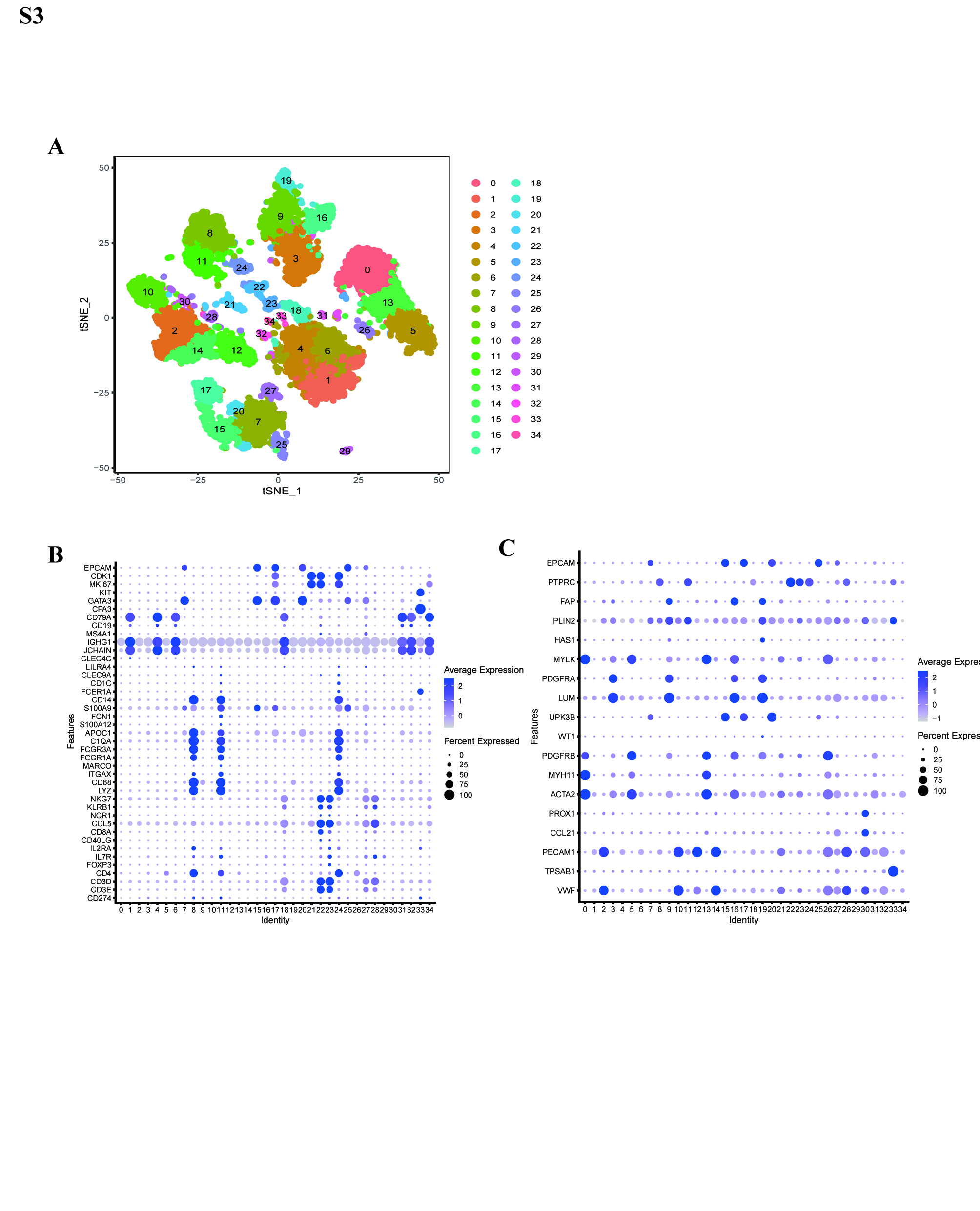

Supplement: Supplementary file 3 [file Image3.tif]
